# The Application of Dynamic Models to the Exploration of *β*_1_-AR Overactivation as a Cause of Heart Failure

**DOI:** 10.1155/2018/1613290

**Published:** 2018-07-30

**Authors:** Xiaoyun Wang, Min Zhao, Xiaoqiang Wang, Shuping Li, Ning Cao, Huirong Liu

**Affiliations:** ^1^College of Mathematics, Taiyuan University of Technology, Taiyuan, Shanxi, China; ^2^Department of Scientific Computing, Florida State University, Tallahassee, FL, USA; ^3^Department of Mathematics, North University of China, Taiyuan, Shanxi, China; ^4^Department of Physiology and Pathophysiology, School of Basic Medical Sciences, Capital Medical University, Beijing, China; ^5^Beijing Key Laboratory of Metabolic Disorders Related Cardiovascular Diseases, Capital Medical University, Beijing, China

## Abstract

High titer of *β*_1_-adrenoreceptor autoantibodies (*β*_1_-AA) has been reported to appear in heart failure patients. It induces sustained *β*_1_-adrenergic receptor (*β*_1_-AR) activation which leads to heart failure (HF), but the mechanism is as yet unclear. In order to investigate the mechanisms causing *β*_1_-AR non-desensitization, we studied the beating frequency of the neonatal rat cardiomyocytes (NRCMs) under different conditions (an injection of isoprenaline (ISO) for one group and *β*_1_-AA for the other) and established three dynamic models in order to best describe the true relationships shown in medical experiments; one model used a control group of healthy rats; then in HF rats one focused on conformation changes in *β*_1_-AR; the other examined interaction between *β*_1_-AR and *β*_2_-adrenergic receptors (*β*_2_-AR). Comparing the experimental data and corresponding Akaike information criterion (AIC) values, we concluded that the interaction model was the most likely mechanism. We used mathematical methods to explore the mechanism for the development of heart failure and to find potential targets for prevention and treatment. The aim of the paper was to provide a strong theoretical basis for the clinical development of personalized treatment programs. We also carried out sensitivity analysis of the initial concentration *β*_1_-AA and found that they had a noticeable effect on the fitting results.

## 1. Introduction

The incidence of heart failure (HF) is increasing year by year throughout the world, as is the cost of treating it [1]. Accurately recognising the cardiac signals associated with HF not only would prevent the progress of chronic HF, but also can guide individual treatment and positively influence the normal progression of the disease [2]. A variety of mechanisms are involved in HF progression, including nervous system inhibition. Heart failure itself causes cardiovascular irregularity due to material imbalance and cell damage [3]. Studies have shown that sustained activation of the sympathetic nervous system (SNS) is the core mechanism trigger of heart failure [4]. The main way in which the overactivation of the SNS occurs is the overactivation of the cardiomyocytes at the *β*_1_-adrenergic receptor (*β*_1_-AR) [5]. *β*_1_-adrenergic receptor autoantibodies (*β*_1_-AA) were found in the serum of patients with dilated cardiomyopathy in a study [6]. As *β*_1_-AA can bind with and activate *β*_1_-AR, it has a *β*_1_-AR agonist-like effect [7]. Excessive activation of *β*_1_-AR leads to impaired cardiac function [8]. A study found that, in addition to catecholamine *β*_1_-AR agonist ISO, *β*_1_-adrenoceptor (*β*_1_-AA) autoantibodies that could cause continuous activation of *β*_1_-AR [9] were detected in 40%–60% of HF patients [8]. *β*_1_-AR and *β*_2_-AR (*β*_1_-adrenergic receptors) link through the C-terminal, which are within cardiac cells, and belong to the G protein-coupled receptor family [10]. *β*_1_-AR and *β*_2_-AR can form heterodimers [11], where *β*_2_-AR downstream couples with stimulatory G protein (*G*_*s*_) and inhibitory G protein (*G*_*i*_) [12]. Another study [13] found that, in the early stages of heart failure, when *β*_1_-AR is activated, it can cause the *β*_2_-AR downstream signal to be converted from *G*_*s*_ to *G*_*i*_, and *G*_*i*_ is activated by the activity of G protein-coupled receptor kinase 2 (GRK2), which then causes *β*_1_-AR phosphorylation, as well as *β*-arrestin binding to form endocytosis, after which *β*_1_-AR endocytosis [13] inhibits the overactivation of *β*_1_-AA. There are two main mechanisms involved in the continuous activation of *β*_1_-AR: *β*_1_-AR conformation changes and interaction between *β*_1_-AR and *β*_2_-AR. However, the mechanisms for how *β*_1_-AA induces continuous activation of *β*_1_-AR are not entirely clear.

To identify the core molecular mechanism of *β*_1_-AR non-desensitization, we adopted a novel approach by establishing differential dynamic models. We used *C*_++_ to estimate the parameters, obtained modified 60-minute snapshots highlighting the experimental data, and made predictions on how the images would progress over the next 60 minutes. Then we took advantage of AIC to select the optimal model of *β*_1_-AR non-desensitization induced by *β*_1_-AA, and the corresponding mechanism. *β*_2_-AR plays an important role in the activation of *β*_1_-AR, which can be used to estimate cardiac function, prevent deterioration of cardiac function, and improve the quality of life of the patients with HF.

This paper is organized as follows. In Section 2, we established the models and listed the differential equations separately.  Section 3 focused on the analysis of the models and determination of the parameters. We select the optimal model to identify the key molecular mechanism, by applying the Akaike information criterion (AIC). Results on the sensitivity analysis of parameters are given in Section 4.  Section 5 is the conclusion.

**Figure 1:**
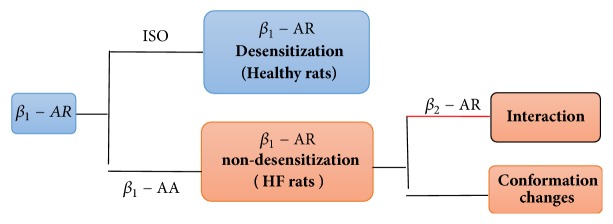
Relation diagram among the ISO, *β*_1_-AA, *β*_1_-AR, and *β*_2_-AR.

Figure 1 shows the interrelationships between the various substances. It reflects the receptor desensitization when the *β*_1_-AR is combined with ISO and non-desensitization when the *β*_1_-AR is combined with *β*_1_-AA, causing conformational changes and interaction between the *β*_1_-AR and the *β*_2_-AR, eventually leading to HF.

## 2. Methods

### 2.1. Extraction and Detection of Neonatal Rat Cardiomyocytes

Laboratory animal medicine of Capital Medical University provided 20 male newborn rats born 0–3 days as raw materials for extracting neonatal rat cardiomyocytes. According to previous method [14], the details of myocardial cell isolation and culture of neonatal rats are as follows: (1) open the sternum, expose the heart, clip it with tweezers, and wash in cold phosphate-buffered saline (PBS); (2) remove excess connective tissue from the washed heart and cut with ophthalmic scissors; (3) the heart fragments were aspirated into a centrifuge tube, and cold PBS was added and then centrifuged at 1000 *rpm* for 5 *mins*; (4) remove the centrifuge tube and vacuum suction PBS; (5) add 1 *ml* of 0.25 % trypsin and 1 *ml* of 0.25 % collagenase, blow vigorously, set in a 37°*C* water bath, and shake for 20 *mins*; (6) add 1 *ml* fetal bovine serum to stop digestion; (7) digestion was collected by adding 2 *ml* of DMEM containing 10% fetal bovine serum (FBS), 1000 *rpm*, and after centrifugation for 10 *mins*, the supernatant was discarded and fresh medium was added for differential adherence; (8) after adherence for 1 *h*, the culture medium was transferred to a new centrifuge tube and centrifuged at 1000 *rpm* for 5 *mins*. Cells were collected and the supernatant was discarded. Fresh medium was added and transferred to a 6-well plate for cell culture and 2 *ml* of DMEM low-glucose medium was added to each well. Detection of the beating frequency of NRCMs [15] was as follows: the culture medium was replaced on the day of the experiment and stably incubating in a 37°*C* cell culture incubator for 30 minutes, the 6-well plate was placed on a constant-temperature table of inverted microscopy, and a total of 10 fields of view of 3 six-well plates were randomly observed. Each field was measured for 30 seconds at a time, and the number of synchronized contractions of an isolated single cell or a group of cardiomyocytes in the untreated group was measured.

Then, we used 0.1*μM* of ISO, *β*_1_-AA, and IgG (immunoglobulin G) (eliminating the effects of *β*_1_-AA itself) to stimulate, respectively. After that, the beating frequency of NRCMs was measured by Live Cell Imaging System offered by Medical Sciences Center Lab of Capital Medical University. Specifically, we measured the frequency in beat per minute of the cardiomyocytes after stimulation by live cell workstation and visualization at 63-fold magnification. Before being exposed to drugs, the cells had been stabilized for 10 *mins* in the system. The present study complies with the recommendations in the Guide for the Care and Use of Laboratory Animals protocol, NIH guidelines (Guide for the Care and Use of Laboratory Animals), and conformed to AVMA Guidelines on Euthanasia.

Tables 1 and 2 showed the measured data of beating frequency of NRCMs added to ISO and *β*_1_-AA. We used the beat frequency of NRCMs at time 0 as the base value, which was recorded as 0. When the standard deviation was greater than or equal to 4, the data was appropriately adjusted within the scope of standard deviation.

**Figure 2:**
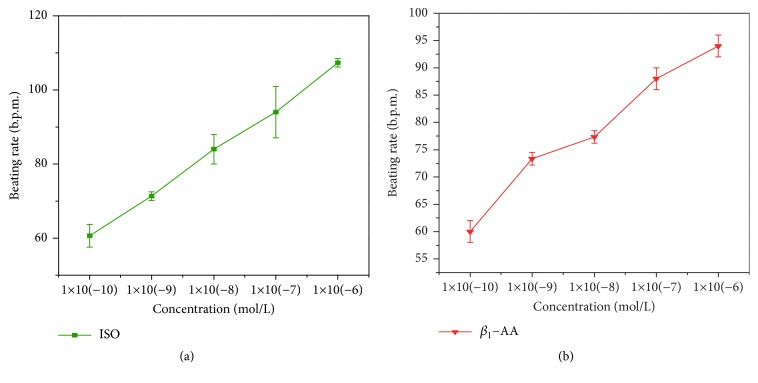
Relationship between different reactant concentrations and beating frequency of NRCMs.** (a)** Control model in three minutes.** (b)** Experiment model in three minutes. Multiple sets of experiments were performed using the previously mentioned method for detecting NRCMs, and the beating frequency of NRCMs at a concentration of 0.1 *nM* to 1 *μM* was measured.

Figure 2 reflects the positive correlation between concentration and beating frequency of NRCMs. Also, Martinsson et al. [16] studied the relationship between ISO and heart rate in their experiment results.

### 2.2. The Models

There are many molecular mechanisms causing *β*_1_-AR non-desensitization, among which conformation changes and interaction are the most likely ones. In order to clarify the most likely molecular mechanism, we established dynamical models including control model and experiment models to study the specific molecular mechanisms.

#### 2.2.1. Explanation of Interaction

**Definition 1 (a protein-to-protein interaction (*PPI*) [17]).**

Proteins rarely act alone as their functions tend to be regulated. Many molecular processes within a cell are carried out by molecular machines that are built from a large number of protein components organized by their *PPIs* (protein-protein interactions). In the experimental groups, *β*_1_-AR and *β*_2_-AR belong to the G protein-coupled receptor family that are connected through the C-terminus [10] which belongs to PPI. Although *β*_1_-AA does not directly bind to *β*_2_-AR, *β*_1_-AA will indirectly affect the conformation of *β*_2_-AR by activating *β*_1_-AR based on the studies of laboratory animal medicine of Capital Medical University. Therefore, *β*_1_-AA can interfere with their dimerization which reflects the fact that *β*_2_-AR can affect the persistence of *β*_1_-AR activation.

#### 2.2.2. The Establishment of the Model Block Diagrams

**Figure 3:**
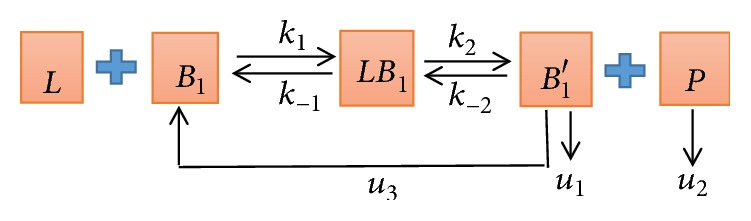
The block diagram of control model. ISO (L) activates *β*_1_-AR (*B*_1_) receptor generating intermediate complexes *LB*_1_ causing endocytosis of *β*_1_-AR, so that it can no longer come into contact with the protected ligand; then the complexes LB produce a new substance P and structurally changed receptor *β*_1_-AR (*B*_1_′) that returns to the cell surface to terminate the signal.

**Figure 4:**
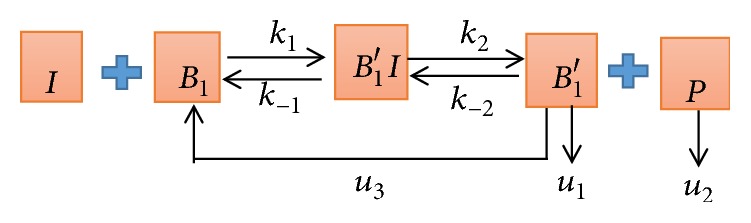
The block diagram of conformation changes model. *β*_1_-AA (I) activates *β*_1_-AR (*B*_1_) receptor generating intermediate complexes *B*_1_′*I* with conformation changes. The production of *B*_1_′*I* continues to activate the signal, and then it produces a new substance P and structurally changed receptor *β*_1_-AR (*B*_1_′).

**Figure 5:**
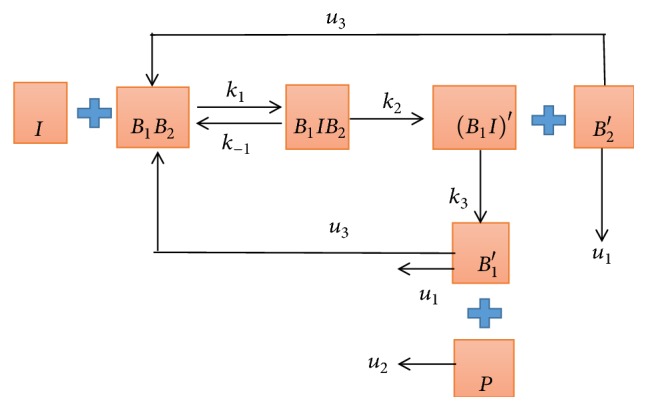
The block diagram of interaction model. We also take *β*_2_-AR (*B*_2_) into consideration in the reaction based on medical experiments. According to the studies of laboratory animal medicine of Capital Medical University, they found that *β*_1_-AA had a unique characteristic that can inhibit heterodimerization of *β*_1_/*β*_2_-AR and thus lead to decompose of *β*_1_-AR and *β*_2_-AR that are originally connected, resulting in endocytosis inhibition and sustained activation of the signal. Therefore, there is no conjugation of *β*_1_-AR and *β*_2_-AR (*B*_1_*B*_2_) in the products. Besides, *β*_1_-AA (I) cannot directly bind to *β*_2_-AR (*B*_2_), so there is no *IB*_2_ in the products. Thus, we consider that *B*_1_*IB*_2_ (I and *B*_2_ are combined to *B*_1_) are intermediate complexes and *B*_1_′*I*, *B*_2_′ are the products with structure change.

The possible molecular mechanisms about *β*_1_-AR overactivation are conformation changes and interaction. To determine a more specific molecular mechanism, we propose the dynamical models including control model and experiment models, where experiment models are composed of conformation changes model and interaction model. In order to facilitate the study and simplify the reaction diagram, we use corresponding letters instead of reactants and the definition of the corresponding parameters in dynamical models; see Table 3.

(*1) The Block Diagram of Control Model (See Figure 3)*. In Figure 3, *k*_1_ represents the combined velocity of L and *B*_1_, *k*_−1_ represents the reverse reaction velocity, *k*_2_ represents the velocity of *LB*_1_ decomposition, *k*_−2_ is the reverse reaction velocity, and *u*_1_ and *u*_2_ represent the degradation velocity of *B*_1_′ and P, respectively. *u*_3_ represents the velocity at which undegraded *B*_1_′ returns to the cell surface and participates in the reaction again.

(*2) The Block Diagram of Conformation Changes Model (See Figure 4)*. In Figure 4, *k*_1_ represents the combined velocity of I and *B*_1_, *k*_−1_ represents the reverse reaction velocity, *k*_2_ represents the velocity of *B*_1_′*I* decomposition, *k*_−2_ is the reverse reaction velocity, and *u*_1_ and *u*_2_ represent the degradation velocity of *B*_1_′ and P, respectively. *u*_3_ represents the velocity at which undegraded *B*_1_′ returns to the cell surface and participates in the reaction again.

(*3) The Block Diagram of Interaction Model (See Figure 5)*. In Figure 5, *k*_1_ represents the combined velocity of I and *B*_1_*B*_2_, *k*_−1_ represents the reverse reaction velocity, *k*_2_ represents the velocity of *B*_1_*IB*_2_ decomposition, *k*_3_ represents the velocity of (*B*_1_*I*)′ decomposition, and *u*_1_ and *u*_2_ represent the degradation velocity of *B*_1_′ and P, respectively. *u*_3_ represents the velocity at which undegraded *B*_1_′ returns to the cell surface and participates in the reaction again. Also, *β*_1_-AR and *β*_2_-AR belong to the G protein-coupled receptor family [10], and thus we assume that they have the same degradation velocity *u*_1_.

Next, we established three mathematical models as shown in Figures 3, 4, and 5 and established the corresponding ordinary differential equations based on the relevant theoretical knowledge of biochemical reaction models in cell and molecular biology [18, 19].

#### 2.2.3. Corresponding Differential Equations

(*1) The Control Model*. we propose a control model for healthy rats based on Figure 3. The time evolution of control model is described by five coupled differential equations.

(1) dLdt=−k1LB1+k−1LB1,dB1dt=−k1LB1+k−1LB1+u3B1′,dLB1dt=k1LB1−k−1LB1−k2LB1+k−2B1′P,dB1′dt=k2LB1−u1B1′−u3B1′−k−2B1′P,dPdt=k2LB1−k−2B1′P−u2P,

where the initial conditions of the control model are [*L*](0) = [*L*_0_] = 1 × 10^−7^*mol*/*L*, [*B*_1_](0) = [*B*_10_] = 3 × 10^−8^*mol*/*L*, [*LB*_1_](0) = 0, [*B*_1_′](0) = 0, and [*P*](0) = 0.

(*2) The Conformation Changes Model.* We have known that *β*_1_-AA and ISO compete for different binding sites of *β*_1_-AR, which belongs to the competitive inhibition in different positions of *β*_1_-AR [10]. Assuming that there is no reaction between *β*_1_-AA and *β*_2_-AR, the time evolution of conformation changes model based on Figure 4 is described by five coupled differential equations.

(2) dIdt=−k1IB1+k−1B1′I,dB1dt=−k1IB1+k−1B1′I+u3B1′,dB1′Idt=k1IB1−k−1B1′I−k2B1′I+k−2B1′P,dB1′dt=k2B1′I−k−2B1′P−u1B1′−u3B1′,dPdt=k2B1′I−k−2B1′P−u2P,

where the initial conditions of the conformation changes model are [*I*](0) = [*I*_0_] = 1 × 10^−7^*mol*/*L*, [*B*_1_](0) = [*B*_10_] = 2.3 × 10^−9^*mol*/*L*, [*B*_1_′*I*](0) = 0, [*B*_1_′](0) = 0, and [*P*](0) = 0.

(*3) The Interaction Model*. In the preliminary study, *β*_1_-AR and *β*_2_-AR form heterodimer through the C-terminal [10], while *β*_1_-AA can be combined with *β*_1_-AR but not directly with *β*_2_-AR [15]. Thus, we consider the effect of *β*_2_-AR on the experimental group to establish interaction model, while the other conditions of the experiment group remain unchanged.

(3) dIdt=−k1IB1B2+k−1B1IB2,dB1B2dt=−k1IB1B2+k−1B1IB2+u3B1′+u3B2′,dB1IB2dt=k1IB1B2−k−1B1IB2−k2B1IB2,dB1I′dt=k2B1IB2−k3B1I′,dB1′dt=k3B1I′−u1B2′−u3B1′,dB2′dt=k2B1IB2−u1B2′−u3B2′,dPdt=k3B1I′−u2P,

where the initial conditions of the interaction model are [*I*](0) = [*I*_0_] = 1 × 10^−7^*mol*/*L*, [*B*_1_*B*_2_](0) = 1.8 × 10^−9^*mol*/*L*, [*B*_1_*IB*_2_](0) = 0, [(*B*_1_*I*)′](0) = 0, [*B*_1_′](0) = 0, [*B*_2_′](0) = 0, and [*P*](0) = 0.

Next, we will use second order Runge-Kutta method to estimate the parameters of systems (1), (2), and (3) and then select the optimal model of *β*_1_-AR non-desensitization.

## 3. Model Analysis and Results

### 3.1. Parameter Fitting and Graphs Analysis

Second order Runge-Kutta method is used to solve the ODE systems obtained in our models. Our parameter fitting is an optimization process, which minimizes the squared summation of [*AB*]_*pred*_ − [*AB*]_*data*_ at all time [*AB*]_*data*_ got measured, where for simplicity we use [*AB*] to denote the concentrations of the intermediate products *LB*_1_, *B*_1_′*I*, and *B*_1_*IB*_2_ in the three models, respectively (i.e., [*LB*_1_], [*B*_1_′*I*], and [*B*_1_*IB*_2_]). We also denote the total squared summation by *S*. Obviously *S* is a function of all the parameters that require fitting. Given a set of parameters, second order Runge-Kutta method gives the value of [*AB*]_*pred*_ and thus *S*. We use steepest descent method to find the minimum of *S*. In this process, the gradient of *S* over all parameters is calculated numerically. For example, for positive parameter *k*_*i*_,

(4) $$\begin{array}{c} \frac{\partial S}{\partial k_{i}}=\frac{S\left(k_{i}\left(1+\Delta \right)\right)-S\left(k_{i}\right)}{k_{i}\Delta }. \end{array}$$

In our program Δ = 1.0*e* − 6, and the time step in the Runge-Kutta method is chosen to be 0.01 min.

We use the measured values in Table 1 to do the parameter fitting for the control model and the values in Table 2 for the experiment models. Note that the measured data in the tables is not the exact concentration of [*AB*]. Instead, we assumed that the measured data is proportional to the concentration of [*AB*]. In other words, we introduce an extra parameter *λ*, and [*AB*]_*data*_ = *λT*_*data*_, where *T*_*data*_ is the measured data in the tables. In the optimization process, we trait *λ* in the same way we trait other parameters. And *λ* is also get fitted. Combining Figure 2 and parameter fitting method, we determined that the concentration is directly proportional to the heart rate of NRCMs and the proportionality factor is 0.0025.

**Figure 6:**
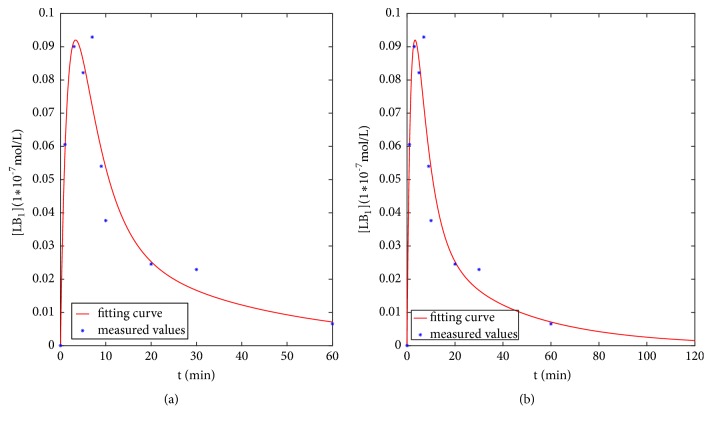
The fitting and prediction graphs of control model.** (a)** Fitting graph.** (b)** Prediction graph at 120 mins.

**Figure 7:**
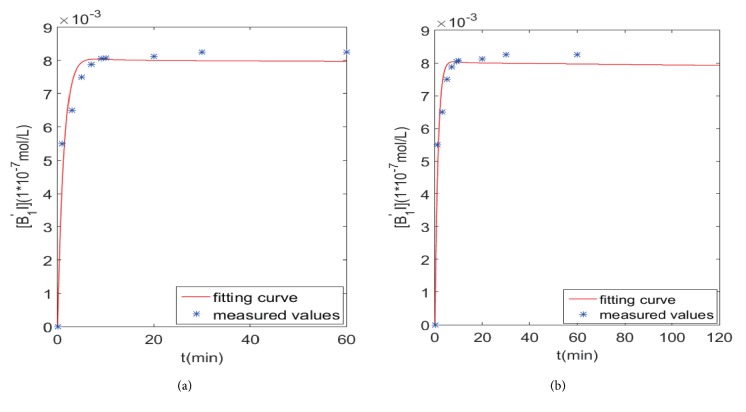
The fitting and prediction graphs of conformation changes model.** (a)** Fitting graph.** (b)** Prediction graph at 120 mins.

**Figure 8:**
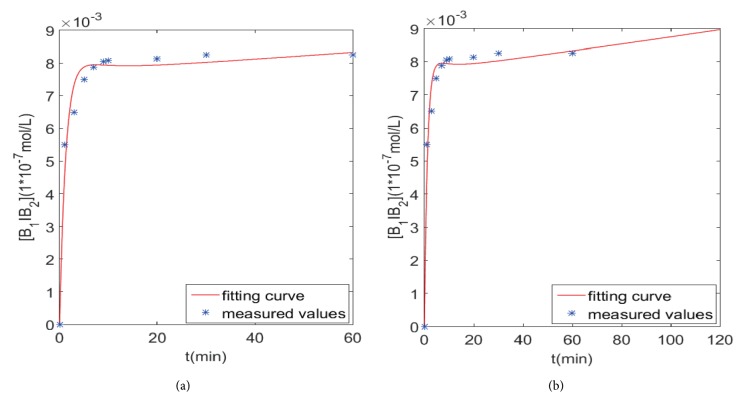
The fitting and prediction graphs of interaction model.** (a)** Fitting graph.** (b)** Prediction graph at 120 mins.

Next, we analyze the fitting of the graphs and the prediction of the different time points, as shown in Figures 6–8.

Figure 6(a) shows the fitting results of control group model from 0 *min* to 60 *mins*, in which the points are experimentally measured and the curve is the solution of dynamical control group model of [*LB*_1_]. And the fitting curve finally declined which reflects the fact that *β*_1_-AR desensitizes when reacting with ISO in healthy rats. However, the situation was quite different with HF rats.

Figure 6(b) displays the prediction result from 60 *mins* to 120 *mins*. It predicts that [*LB*_1_] continues to decrease, eventually tends to zero, and becomes stable, which clearly showed the mechanism of *β*_1_-AR desensitization.

Figure 7(a) presents the fitting results of the conformation changes model from 0 *min* to 60 *mins*, in which the points are experimentally measured and the curve is the solution of dynamical conformation changes model of [*B*_1_′*I*].

Figure 7(b) is the prediction results from 60 *mins* to 120 *mins*. The [*B*_1_′*I*] curve eventually stabilized in (b), which clearly showed the mechanism of *β*_1_-AR non-desensitization.

Figure 8(a) presents the fitting results of the interaction model from 0 *min* to 60 *mins*, in which the points are experimentally measured, the curve is the solution of dynamical interaction model of [*B*_1_*IB*_2_], and the fitting curve finally stabilized.

Figure 8(b) is the prediction results from 60 *mins* to 120 *mins*. The upward trend of the [*B*_1_*IB*_2_] curve reflects the effects of *β*_1_-AA and *β*_2_-AR on the non-desensitization of the *β*_1_-AR.

**Figure 9:**
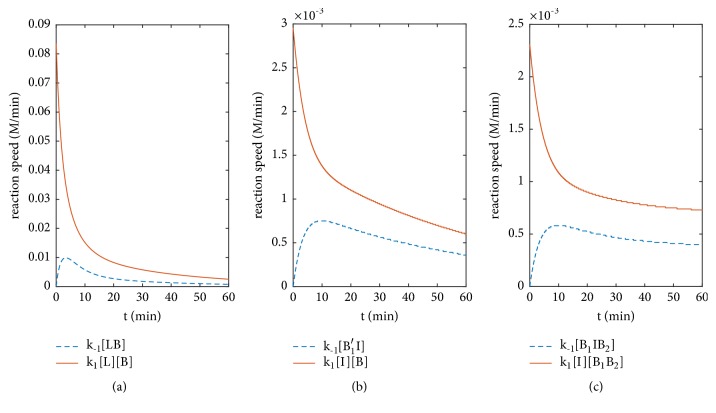
Comparison of speeds of positive and negative reactions between experimental models and control model.** (a)** Control model.** (b)** Conformation changes model.** (c)** Interaction model.

Then, we analyze the reaction speeds and Table 4 lists the reaction velocities of three models. Since the units of positive reaction velocities and reverse reaction velocities are different, they cannot be directly compared. Therefore, we simulated the reaction speeds figures of the control group and the experimental groups, and the reaction speed is the product of the reaction velocity and concentration. See Figure 9.

As can be seen from Figure 9, the positive and negative reaction speeds of the control model and experimental models are greater than those of the reverse reaction, and the positive reaction speeds decrease rapidly. In the course of the decrease of the positive reaction speeds, the speed of reversed reaction continues to rise. Finally, their tendency gradually slows down.

### 3.2. Model Selection

**Akaike's information criterion** [20–22] (AIC) is a measure of the goodness of fit of an estimated statistical model and a tool for model selection. Given a data set, several competing models can be ranked according to their AIC, with the one with the lowest AIC being the best. The AIC is defined as follows:

(5) AIC=nInRe+2k,Re=RSSn,

where RSS is the residual sum of squares, *n* is the amount of the real data, and *k* is the total amount of estimated parameters in the model. AIC_*c*_ is AIC with a second order correction for small sample sizes (*n*/*k* < 40), to start with

(6) $$\begin{array}{c} AIC_{c}=AIC+\frac{2k\left(k+1\right)}{n-k-1}. \end{array}$$

Moreover, AIC difference is defined as Δ_*i*_ = *AIC*_*i*_ − *AIC*_*min*_, where *AIC*_*i*_ is the AIC or AIC_*c*_ of the *i*th model, and *AIC*_*min*_ is the AIC or AIC_*c*_ of the model with minimal AIC. If 0 ≤ Δ_*i*_ ≤ 2, the *i*th model has substantial data support. If 4 ≤ Δ_*i*_ ≤ 7, the *i*th model has considerable less data support. If Δ_*i*_ ≥ 10, the *i*th model has essentially no data support.

Since the *n*/*k* = 10/4 < 40, we use AIC_*c*_ to select the model, which determines the mechanism of the *β*_1_-AR non-desensitization. In order to verify the validity of the models, we apply AIC to view the residuals between theoretic and real data under three models. We calculated the AIC_*c*_ values of the conformation model and interaction model which are, respectively, *AIC*_*c*1_ ≈ −7.287, *AIC*_*c*2_ ≈ −8.494. By comparing the AIC_*c*_ values calculated in the two models above, we conclude easily that *AIC*_*c*1_ > *AIC*_*c*2_, *AIC*_*cmin*_ ≈ −8.494. It is obvious that the possibility of interaction is the largest, which conforms to the analysis of graphs and parameter.

## 4. Sensitivity Analysis

**Figure 10:**
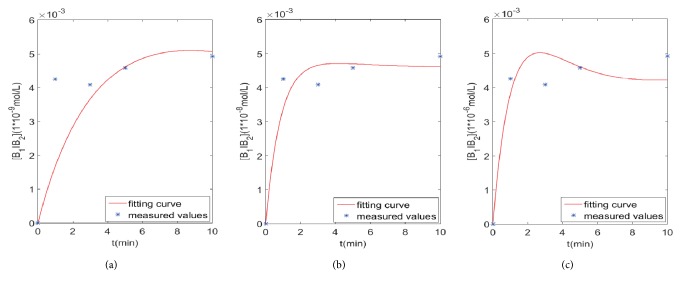
The fitting graphs of [*B*_1_*IB*_2_] with the change of initial concentrations of *β*_1_-AA (I) in interaction model.** (a) **1 × 10^−9^*mol*/*L*.** (b) **1 × 10^−8^*mol*/*L*.** (c) **1 × 10^−6^*mol*/*L*.

We determined that the interaction model between *β*_1_-AR and *β*_2_-AR is the optimal model for heart failure through the AIC criterion. Next, when the initial concentration of *β*_1_-AA is different, we analyzed the figures and data (measured by Capital Medical University) fitting of the interaction model between *β*_1_-AR and *β*_2_-AR. From Figure 10, we can see that the initial concentration of *β*_1_-AA has a certain effect on the fitting result.  Table 5 shows the reaction velocity for the interaction model at different concentrations.

Figure 10, respectively, showed the graphs of [*B*_1_*IB*_2_] when the initial concentrations of *β*_1_-AA are 1 × 10^−9^*mol*/*L*, 1 × 10^−8^*mol*/*L*, and 1 × 10^−6^*mol*/*L* in 10 minutes, while the concentration of *B*_1_*B*_2_ remains unchanged at 1.8 × 10^−9^*mol*/*L*. And we can see that when the concentration of *β*_1_-AA is 1 × 10^−9^*mol*/*L*, the fitting result is the worst, followed by 1 × 10^−6^*mol*/*L* and 1 × 10^−8^*mol*/*L*. As can be seen from Figure 8, when concentration of *β*_1_-AA is 1 × 10^−7^*mol*/*L*, the fitting result is the best. Therefore, the optimum concentration of mice monoclonal *β*_1_-AA leading to the NRCMs beating frequency was 0.1 *μM* (detected from 1 *nM* to 1 *μM*).

## 5. Conclusion

We analyzed the two types of mechanisms that cause HF; it can be concluded that conformation changes and interactions between *β*_1_-AR and *β*_2_-AR both contribute to the HF progression in rats and the interaction has the greater impact on it. In our study, for the first time, the model block diagrams described the causation of HF. By comparing the experimental group models with the control group model and then calculating the AIC_*c*_, we concluded that the interaction model between *β*_1_-AR and *β*_2_-AR was the optimal model, which leads to the important role of *β*_2_-AR in the progression of HF. *β*_2_-AR and *β*_1_-AR connect together and participate in a reaction, which then causes conformation changes in receptor *β*_1_-AR. This results in endocytosis inhibition, which ultimately leads to *β*_1_-AR non-desensitization and HF. Thus, *β*_2_-AR is an important consideration in guiding the treatment of HF.

Previous studies have found that *β*_1_-AR and *β*_2_-AR could form heterodimers, although they also found that the autoantibody *β*_1_-AA did not bind to *β*_2_-AR [15]. We found that *β*_1_-AA could inhibit heterodimerization of *β*_1_/*β*_2_-AR, observing laboratory experiments on animals at Capital Medical University. Therefore, we speculated that *β*_1_-AA might indirectly affect the *β*_2_-AR- C-terminal conformation and interfere with heterodimerization of *β*_1_/*β*_2_-AR. This would lead to *β*_1_-AR endocytosis inhibition, to continuous signal activation, and ultimately to heart failure.

Because the interaction between *β*_1_-AR and *β*_2_-AR was found to be the trigger mechanism of heart failure progression, receptor *β*_2_-AR should be further studied to determine if it could play a role in heart failure treatment. In the presence of *β*_1_-AA-positive heart failure, *β*_2_-AR can be used as a drug and therapeutic target to improve the interaction between *β*_1_-AR and *β*_2_-AR, thereby decreasing the risk of further heart failure.

## Figures and Tables

**Table 1:** Data on frequency of beats of measured NRCMs added to ISO.

Time (*min*)	Mean values (*bmp*)	Standard Deviation (*bmp*)
	n=3	
0.0	0	3.055
1.0	24.667	5.292
3.0	36.667	3.464
5.0	33.46	7.211
7.0	37.837	4.163
9.0	22.0	4.163
10.0	15.333	5.774
20.0	10.0	1.155
30.0	9.333	3.055
60.0	2.667	4.0

**Table 2:** Data on frequency of beats of measured NRCMs added to *β*_1_ − *AA*.

Time (*min*)	Mean values (*bmp*)	Standard Deviation (*bmp*)
	n=3	
0.0	0.0	2.0
1.0	22.0	2.0
3.0	26.0	2.0
5.0	30.0	3.464
7.0	31.5	7.024
9.0	32.2	5.774
10.0	32.33	4.619
20.0	32.5	7.211
30.0	33.0	5.292
60.0	33.0	6.0

**Table 3:** Nonstandard abbreviations.

**L**	**ISO**	**P**	**product**
*B*_1_	*β*_1_−**AR**	**L** *B*_1_	**intermediate complexes**
*B*_1_′	*β*_1_−**AR with structure changes**	*B*_1_′*I*	**intermediate complexes**
*B*_2_	*β*_2_−**AR**	*B*_1_ *B*_2_	**conjugation of ** *β*_1_−**AR and***β*_2_−**AR**
*B*_2_′	*β*_2_−**AR with structure changes**	(*B*_1_*I*)′	**the product with structure change**
**I**	*β*_1_−**AA**	*B*_1_ *IB*_2_	**intermediate complexes**

**Table 4:** Velocities in three models.

models	*k*_1_	*k*_−1_	*k*_2_	*k*_−2_	*k*_3_	*u*_1_	*u*_2_	*u*_3_
	*M* ^−1^ *min* ^−1^	*min* ^−1^	*min* ^−1^	*M* ^−1^ *min* ^−1^	*min* ^−1^	*min* ^−1^	*min* ^−1^	*min* ^−1^
control model	0.279	0.107	0.273	1*∗*10 ^−6^	–	0.039	0.011	0.028
conformation								
changes model	0.275	0.474	0.018	0.021	–	1*∗*10^−6^	0.100	0.271
interaction model	0.361	0.422	0.014	–	0.153	0.122	0.100	0.191

**Table 5:** Velocity values in interaction model.

Concentration of *β*_1_-AA	*k*_1_	*k*_−1_	*k*_2_	*k*_3_
(*mol*/*L*)	(*M*^−1^*min*^−1^)	(*min*^−1^)	(*min*^−1^)	(*min*^−1^)
1 × 10^−9^	11.173	0.000001	0.067	0.000001
1 × 10^−8^	3.201	0.753	0.059	0.408
1 × 10^−6^	0.035	0.362	0.343	0.238

## Data Availability

The study conformed to AVMA Guidelines on Euthanasia and the Guide for the Care and Use of Laboratory Animals protocol published by the Ministry of Education of People's Republic of China. All studies were approved by Capital Medical University Committee on Animal Care. According to the data provided by Capital Medical University, we extracted the original data and established the relationship between experimental data and mathematical models to facilitate our research.

## Supplementary Materials

Supplementary 1 The picture in the supplementary material reflects the increased beating frequency of NRCMs stimulated with different concentrations of *β*_1_-AA. As can be seen, 0.1 *μM*  *β*_1_-AA is the optimal concentration for the increase of beating frequency in NRCMs, which is consistent with the results of the sensitivity analysis.

Supplementary 2 The video file in the supplementary material mainly indicates that we successfully extracted NRCMs from the experiments and measured the beating frequency.
