# Supplementary figures and images for: The Application of Dynamic Models to the Exploration of β1-AR Overactivation as a Cause of Heart Failure
Source: Comput Math Methods Med. 2018 Jul 30;2018:1613290. doi: 10.1155/2018/1613290 (PMC6091447; doi:10.1155/2018/1613290)

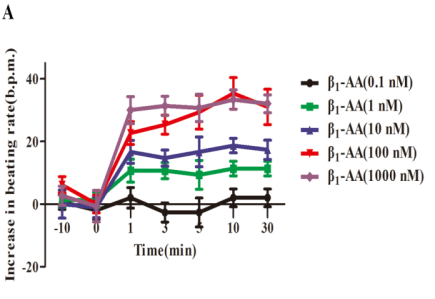

Supplement: Supplementary 1 — The picture in the supplementary material reflects the increased beating frequency of NRCMs stimulated with different concentrations of β1-AA. As can be seen, 0.1 μM β1-AA is the optimal concentration for the increase of beating frequency in NRCMs, which is consistent with the results of the sensitivity analysis. [file 1613290.f1.png]
